# A High-Fat Western Diet Attenuates Intestinal Changes in Mice with DSS-Induced Low-Grade Inflammation

**DOI:** 10.1093/jn/nxab401

**Published:** 2021-12-02

**Authors:** Dimitrios Papoutsis, Sérgio Domingos Cardoso da Rocha, Anne Mari Herfindal, Siv Kjølsrud Bøhn, Harald Carlsen

**Affiliations:** Faculty of Chemistry, Biotechnology and Food Science, Norwegian University of Life Sciences, Ås, Norway; Faculty of Chemistry, Biotechnology and Food Science, Norwegian University of Life Sciences, Ås, Norway; Faculty of Chemistry, Biotechnology and Food Science, Norwegian University of Life Sciences, Ås, Norway; Faculty of Chemistry, Biotechnology and Food Science, Norwegian University of Life Sciences, Ås, Norway; Faculty of Chemistry, Biotechnology and Food Science, Norwegian University of Life Sciences, Ås, Norway

**Keywords:** dextran sodium sulfate, gut inflammation, intestinal microbiota, low-fat diet, Western diet

## Abstract

**Background:**

A Western diet (WD) is associated with increased inflammation in the large intestine, which is often ascribed to the high dietary fat content. Intestinal inflammation in rodents can be induced by oral administration of dextran sodium sulfate (DSS). However, most studies investigating effects of WD and DSS have not used appropriate low-fat diets (LFDs) as control.

**Objectives:**

To compare the effects of a WD with those of an LFD on colon health in a DSS-induced low-grade colonic inflammation mouse model.

**Methods:**

Six-week-old male C57BL/6JRj mice were fed an LFD (fat = 10.3% energy, *n* = 24) or a WD (fat = 41.2% energy, *n* = 24) for 15 wk [Experiment 1 (Exp.1)]. Half the mice on each diet (*n* = 12) then received 1% DSS in water for 6 d with the remainder (*n* = 12 in each diet) administered water. Disease activity, proinflammatory genes, inflammatory biomarkers, and fecal microbiota (16S rRNA) were assessed (Exp.1). Follow-up experiments (Exp.2 and Exp.3) were performed to investigate whether fat source (milk or lard; Exp.2) affected outcomes and whether a shift from LFD to WD 1 d prior to 1% DSS exposure caused an immediate effect on DSS-induced inflammation (Exp.3).

**Results:**

In Exp.1, 1% DSS treatment significantly increased disease score in the LFD group compared with the WD group (2.7 compared with 0.8; *P* < 0.001). Higher concentrations of fecal lipocalin (11-fold; *P* < 0.001), proinflammatory gene expression (≤82-fold), and *Proteobacteria* were observed in LFD-fed mice compared with the WD group. The 2 fat sources in WDs (Exp.2) revealed the same low inflammation in WD+DSS mice compared with LFD+DSS mice. Finally, the switch from LFD to WD just before DSS exposure resulted in reduced colonic inflammation (Exp.3).

**Conclusions:**

Herein, WDs (with milk or lard) protected mice against DSS-induced colonic inflammation compared with LFD-fed mice. Whether fat intake induces protective mechanisms against DSS-mediated inflammation or inhibits establishment of the DSS-induced colitis model is unclear.

## Introduction

Western-type diets are characterized by a high content of saturated fat, cholesterol, and refined sugars and are low in dietary fiber. They are associated with inflammation, both systemically and in the gastrointestinal tract (1–3). Although inflammation generally constitutes a central process of the host's innate immune system, chronic inflammation can initiate pathological conditions. Even a modest increase in inflammatory status (low-grade inflammation) experienced over time, can drive the development of many diseases such as metabolic syndrome, obesity, nonalcoholic fatty liver disease, cardiovascular disease, and cancer (4, 5).

In the gastrointestinal tract, a low-grade inflammation is frequently present and defined as a state of higher inflammatory tonus in mucosal tissue of both small intestine and colon, even though not necessarily manifesting clear pathology (6). Low-grade intestinal inflammation can lead to impaired gut barrier integrity. This can result in leakage of bacterial endotoxins, such as LPSs, as well as other metabolites, and can induce both local and systemic responses (7). It has been shown in animal studies that high-fat Western diets (WDs) can induce or exacerbate intestinal inflammation (8). Specifically, ingestion of fat-rich diets can increase the presence of LPSs systemically (9) and diminish expression of genes related to tight junction proteins in epithelial cells, thereby increasing intestinal permeability (10). The gut microbiota is also affected by high-fat diets (HFDs) and changes can promote an inflammatory status in the host (11). Hallmarks of the effect of a WD on microbiota composition in both humans and mice are decreased bacterial richness (12), increased *Firmicutes*/*Bacteroidetes* ratio (13, 14), and higher abundance of Gram-negative bacteria (15), mainly belonging to the *Proteobacteria* phylum. The low content of dietary fiber in WD has been suggested to be a main driver of microbiota changes with adverse effects on colon health (16, 17). However, a high fat content per se is also suggested as being crucial for the negative effects of a WD (18, 19).

Dextran sodium sulfate (DSS), a synthetic sulfated polysaccharide, is widely used for inducing colitis in rodent models because the induced pathogenesis resembles features of inflammatory bowel disease found in humans (20). DSS-induced inflammation primarily affects the colon through a poorly defined mechanism. DSS concentrations ranging from 2.5% to 5%, either in drinking water or in food, are sufficient to cause an inflamed gut in mouse models (21). Most studies have demonstrated that HFDs, particularly those rich in saturated fats, worsen the colonic effects of DSS, both in DSS-induced colitis mouse models and in cancer models where DSS is combined with the carcinogen azoxymethane (AOM) (22–26). In a study by Lee and coworkers (27) HFD-fed mice manifested aggravated experimental colitis compared with mice following a standard fiber-rich, unpurified rodent diet after DSS exposure. This was shown by more severe histological changes in the colon, decrease of goblet cells, disruption of gut barrier, and alterations of intestinal microbiota. Benninghoff et al. (28) showed that AOM/DSS-induced colorectal cancer was exacerbated with a diet that mimicked an extreme version of a WD (reduced amounts of micronutrients in addition to high concentrations of fat and refined sugar). However, when the same diet was used, but with micronutrients matched to the control diet, they observed no differences in tumorigenesis or inflammation when compared with a low-fat control diet. Therefore, the effect of an HFD on induced colonic inflammation is not fully clear.

Previous studies reporting effects of WD or HFD on inflammation in mice have used high doses of DSS (2–5%) to induce inflammation (24, 29, 30). However, others have demonstrated that a lower concentration of DSS (1% DSS) results in a subclinical inflammatory state with few or no visible signs of intestinal damage and with a moderate induction of proinflammatory genes (31, 32). This is relevant for a number of clinical conditions including inflammatory bowel disease when in remission (33) and irritable bowel syndrome (34). In addition to using high concentrations of DSS, most other studies have also used low-fat control diets, which were poorly matched with regard to fiber content (23, 35, 36). In standard rodent maintenance diet (unpurified diet), commonly used as control, fiber content is higher and more diverse than synthetic experimental rodent diets high in fat. In our experiments, we used a low-fat control diet with fiber content equal to the WD (7% cellulose).

Previous studies investigating the relative effects on colonic inflammation of HFDs compared with low-fat diets (LFDs) have provided inconsistent results. The aim in this study was therefore to conduct a series of experiments to elucidate whether a high-fat WD impacted more adversely colonic inflammation compared with a properly controlled LFD. We hypothesized initially that a WD would exacerbate colonic inflammation more than an LFD. We further hypothesized that both fat source and timing of the high-fat feeding in relation to DSS treatment would influence the outcome.

## Methods

### Animals and diets

#### Experiment 1

Six-week-old male C57BL/6JRj mice (*n* = 48) were purchased from JANVIER LABS and housed in ventilated cages (4 mice per cage) under controlled conditions (12-h light-dark cycle; 25 ± 2°C; 45–55% humidity). After 2 wk of acclimatization with a regular mouse maintenance diet (7.4% fat, 75.1% carbohydrate, 17.5% protein; RM1; Special Diets Services), mice were randomly allocated to 4 experimental groups in a 2 × 2 factorial design (*n* = 12 for each group): *1*) LFD, *2*) LFD+DSS, *3*) WD, and *4*) WD+DSS. The experimental diets were purchased from Research Diets: an LFD (D1404270, 10.3% energy from milk fat) and a WD (D12079B, 41.2% of total energy from milk fat). The diets were matched in terms of protein (casein 15.2% of energy), fiber (7% cellulose), and micronutrients. The difference apart from fat content was that the carbohydrate content (74.5% of energy) in the LFD was primarily maltodextrin and corn starch. Corn starch was partially replaced by sucrose as the main carbohydrate source in the WD. Also, 1.5 g/kg cholesterol was added to the WD but not in the LFD. Combined with naturally occurring cholesterol in milk fat, the WD contained ∼2 g/kg (0.2%) cholesterol. Detailed description of the diets is found in **Supplemental Table 1**. After 15 wk on a WD or LFD, 24 mice (groups 2 and 4) received 1% DSS in their drinking water for 6 d whereas the rest received water.

#### Experiment 2

To test the effects of 2 different types of fat in WD (milk and lard), 32 mice were allocated to the following groups (*n* = 8); *1*) LFD, *2*) LFD+DSS, *3*) WD_milk fat_ +DSS and *4*) WD_lard fat_ +DSS. Housing and acclimatization conditions for both Experiment 2 (Exp.2) and Exp.3 were the same as in Exp.1 mentioned above.The feeding trial lasted for 6 wk and then 1% DSS was introduced in the drinking water of groups 2–4 for 6 d. The first 2 groups were used as controls to determine whether the results from Exp.1 could be reproduced. Both WDs (milk- or lard-based) were purchased from Research Diets (Cat no: D12079B) and had the same energy content in all macronutrients including milk fat and lard fat (41.2%). The fatty acid profiles in the 2 types of fat are presented in **Supplemental Table 2**.

#### Experiment 3

To investigate whether the effect of DSS on intestinal health was directly affected by a WD, 18 mice were allocated to 3 groups (*n* = 6)—2 LFD groups and 1 WD group—for 4 wk of feeding. One day before 1% DSS treatment, 1 of the LFD groups was switched to the WD.

All DSS groups were supplied with freshly made 1% DSS in water every second day for 6 d. Animal welfare was evaluated every second day and scored for disease activity according to a score sheet (**Supplemental Table 3**). Food and water were supplied ad libitum. Body weights and food consumption were recorded once per week.

Experimental procedures were approved by the Norwegian Animal Research Authority (Mattilsynet, FOTS ID 14805) in accordance with the guidelines and recommendations of the Federation of European Laboratory Animal Science Associations.

### Sampling

Samples were collected on day 6 of DSS exposure. Initially, whole blood was collected by cardiac puncture following anesthesia by a cocktail of Zoletil Forte (Virbac), Rompun (Bayer), and Fentadon (Eurovet Animal Health) (ZRF; intraperitoneally 0.1 mL ZRF/10 g body weight), with the following active ingredients: zolezepam (32 mg/kg), tiletamine (32 mg/kg), xylazine (4.5 mg/kg), and fentanyl (26 μg/kg). Blood (0.5–1 mL) was drawn into tubes containing ~50 µL NaEDTA (50 mM) as anticoagulant and mice were then killed by cervical dislocation. Blood was centrifuged (6000 × *g*, 10 min, 4°C) to obtain plasma. Colon mucosa was collected by opening the colon longitudinally and kept in RNAlater (Sigma-Aldrich). Fecal pellets were collected from the colon. All samples were stored at –80°C. Due to failure of collecting and processing some of the samples, the number of data points differed occasionally between groups.

### Epithelial barrier permeability

Barrier permeability was measured by using fluorescein isothiocyanate dextran [FITC dextran, 4 kDa (FD4); Sigma-Aldrich), according to Johnson et al. (37). In brief, mice on termination day (Exp.1) were fasted for 4 h before 600 mg/kg FD4 was orally administered. Whole blood was collected by cardiac puncture ∼3 h post FD4 administration. Plasma was obtained as described above and diluted 1:5 in PBS. FITC dextran was determined by fluorescence-spectroscopy (Synergy H4 Hybrid microplate reader, BioTek instruments; 490 nm _Ex_/520 nm _Em_). FITC dextran concentration was calculated using a standard curve based on 5 points of serial dilutions of FITC dextran in control plasma.

### RNA extraction and qRT-PCR

RNA from colonic mucosa samples was extracted with the NucleoSpin RNA/Protein Purification kit (Macherye-Nagel). Because DSS reduces efficiency of both reverse transcriptase and PCR reactions (38, 39), all colon RNA samples were purified using lithium chloride according to Viennois et al. (39).

cDNA synthesis from RNA was performed (**Supplemental Tables 4** and **5**) using iScript cDNA Synthesis Kit (1708891, Bio-Rad), whereas FirePol EvaGreen qPCR Supermix (08-36-00001, Solis BioDyne) was used for the qRT-PCR reaction in a Light Cycler 480 Instrument II (Roche). The parameter settings were: 12 min at 95°C; 40 cycles of 15 s at 95°C followed by 20 s at optimized primer annealing temperature; 20 s at 72°C. LinRegPCR Software (2017.1.0.0) was used to calculate quantification cycle values and primer efficiency (40). Primers used for mRNA expression (Thermo Fisher Scientific) are presented in **Supplemental Table 6**.

### Lipocalin-2 measurement

Mouse Lipocalin-2/NGAL DuoSet ELISA (R&D systems) was used for measuring lipocalin-2 protein (LCN2) from fecal samples collected on day 6 of DSS exposure based on a protocol described earlier (32). Briefly, fecal suspensions were made by vortexing fecal samples (20 min) in PBS containing 0.1% Tween 20 (100 mg feces in 1 mL buffer). Suspensions were centrifuged (13,500 ×*g*, 10 min, 4°C) and supernatants were collected and subjected to analysis. Samples were diluted 20 times (untreated mice) and 20,000 times (DSS-treated). Optical density at 450 nm was determined with a spectrophotometer (SpectraMax M2; Molecular Devices). LCN2 concentration was estimated from a standard curve using 4-parameter logistic curve fit.

### Lipopolysaccharide binding protein measurement

Lipopolysaccharide binding protein (LBP) in plasma was measured with an ELISA assay according to the manufacturer (Biometec). Plasma was obtained at day 6 (termination day). Plasma samples from control mice were diluted 800 times, whereas samples from 1% DSS-treated mice were diluted ∼1500 times. The concentration was measured by optical density as described for Lipocalin-2/NGAL measurements above.

### 16S rRNA gene sequencing

The workflow has been described previously (41). Briefly fecal pellets were placed in 400 μL S.T.A.R buffer (Roche) containing glass beads (Sigma-Aldrich). Samples were processed by FastPrep 96 (1800 rpm, 40 s, 5 min cooling step in between; MP BioMedicals) to lyse cells and centrifuged (15,900 ×*g*, 10 min, 21°C). Supernatants were treated with protease using the Mag Mini LGC kit (LGC Genomics), and KingFisher Flex DNA extraction robot (Thermo Fisher Scientific) for DNA extraction. Because DSS has an inhibitory effect on PCR (39), extracted DNA from fecal samples was diluted 1:4 prior to amplicon PCR (total dilution of 1:100 in the PCR reaction).

After DNA extraction, the 16S rRNA gene was amplified by PCR (“amplicon PCR”) using prokaryote-targeting primers specific for the variable region of V3-V4 of the 16S rRNA gene (25 cycles) (42). Primer sequences and PCR conditions are listed in **Supplemental Tables 7** and **8**. PCR product was purified with AMPure XP (Beckman-Coulter) and 10 further PCR cycles (“index PCR”) were performed (**Supplemental Tables 9** and **10**) resulting in PCR product of ∼594 bp. The sequences of primers in index PCR are shown in **Supplemental Table 11**. All PCR products were qualitatively confirmed by electrophoresis on a 1.5% agarose gel. Quantification of DNA concentrations of index PCR products, and normalization and pooling of these index PCR products were followed by purification of the pooled library with Sera Mag Beads by following the AMPure XP protocol. The pooled library was diluted to 6 pM and sequenced with the MiSeq Reagent Kit V3 (cat. no. MS-102-3003) on the Illumina MiSeq following Illumina's protocol, generating 300-bp paired-end reads that were further paired-end joined and split into their respective samples, quality-filtered using QIIME (Quantitative Insights Into Microbial Ecology) (43), and clustered with 97% identity and higher using the closed-reference usearch algorithm (version 8) (44, 45) against the SILVA database (version 128) (46). To normalize (rarefy) the sequencing data, 6500 sequences per sample were chosen as a cut-off.

### Statistics

Statistical analyses were performed using GraphPad Prism (version 8.3.1 for Windows; GraphPad Software). Data are presented as individual values with group means ± SEM. When necessary, data were log_10_-transformed to achieve stabilized variance and normality, and geometric group mean with geometric SD was applied as the best way to express the center of distribution. Normal distribution was tested using the Shapiro–Wilk normality test. Using the Brown–Forsythe test, following normality testing and possible transformation, it was investigated whether the variation (SD) within the groups (homogeneity of variance) was significantly different. Based on whether normal distribution was achieved or not, parametric and nonparametric models were used respectively. *P* values < 0.05 were considered significant.

In Exp.1, prior to DSS treatment, body weight change and food intake were analyzed by the mixed effects model. In the case of significant interaction (time × diet), data were analyzed for simple main effect of diet within each time point with Bonferroni correction for multiple comparisons. During DSS exposure, body weight changes (Exp.1, Exp.2, and Exp.3) and disease activity index (DAI) (Exp.1) were analyzed using repeated measures 2-factor ANOVA with Geisser–Greenhouse correction. In case of significant interactions (time × diet) we assessed simple main effect of diet for each time point using Tukey or Bonferroni correction for multiple comparisons. Comparisons of untreated and DSS-treated groups were analyzed using 2-factor ANOVA (effects of diet and treatment). When interactions (treatment × diet) were significant we compared all groups with Bonferroni correction for multiple comparisons. If assumptions for ANOVA were not met, comparisons were performed using another suitable approach as specified in figure legends (unpaired *t* test with Welch correction or Mann–Whitney test). Also, in Exp.1 outliers identified by the Rout method, Q = 1% were excluded. In Exp.2 and Exp.3, 1-factor ANOVA was used for the DSS groups followed by Tukey post hoc analysis for the expression of inflammatory genes. Untreated LFD-fed mice in Exp.2 were not included in the statistical analysis.

Analysis of β diversity was conducted in R (version 4.0.0). Weighted UniFrac distances were calculated using QIIME default scripts (core_diversity_analyses.py) and are based on the normalized (rarefied) OUT table. Nonmetric multidimensional scaling of weighted UniFrac distances was performed using the metaMDS function from the vegan package (47) with autotransform = FALSE and try = 100. Global permutational multivariate analysis of variance (PERMANOVA) on weighted UniFrac distances was performed using the adonis function from the vegan package with 999 permutations. Pairwise PERMANOVA was performed by applying the pairwise.perm.manova function from the RVAideMemoire package (48).

For linear discriminant analysis effect size (LEfSe), relative abundances of taxa were used. Software is available at https://huttenhower.sph.harvard.edu/galaxy, with linear discriminant analysis (LDA) score set at 2.0 and *P* ≤ 0.05.

## Results

### Exp.1

#### 1% DSS induced a disease phenotype in LFD-fed mice

**FIGURE 1:**
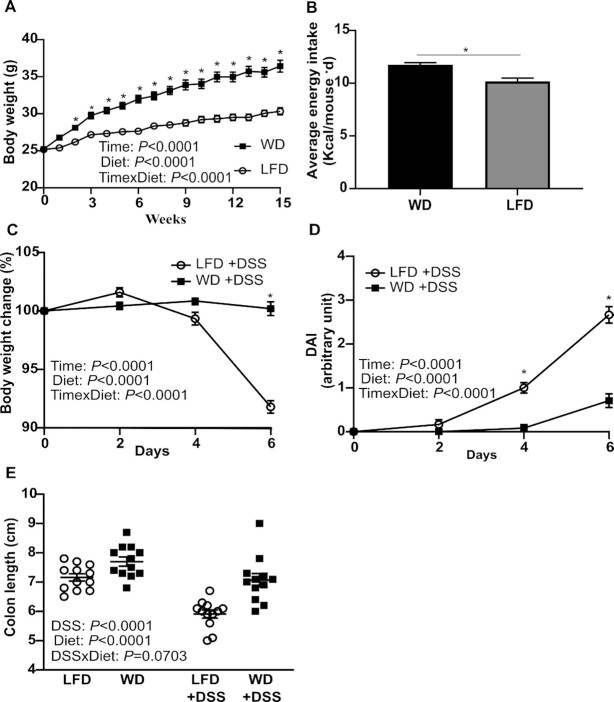
Body weight development and food intake during 15 wk prior to 1% DSS exposure (A, B). Change in body weight (%) measured on days 0, 2, 4, and 6 following start of 1% DSS exposure (C). DAI score for mouse welfare during 1% DSS treatment (D). Colon length from LFD-fed and WD-fed mice with or without 1% DSS (E). Values are means ± SEM (*n* = 12). For panels A, C, and D: *significantly different from LFD at that time, *P* < 0.05. DAI, disease activity index; DSS, dextran sodium sulfate; LFD, low-fat diet; WD, Western diet.

After a feeding period of 15 wk and before administering 1% DSS, weight gain in WD-fed mice was significantly higher compared with LFD-fed mice (**Figure 1**A). Weight gain corroborated with an increased energy intake in the WD group compared with LFD-fed mice (11.7 compared with 10.2 kcal/mouse/d; *P* < 0.01) (Figure 1B).

After 6 d of DSS treatment LFD mice experienced an average 8% weight loss whereas WD-fed mice showed no change in body weight (Figure 1C). In addition, LFD mice had a significantly higher DAI score than WD mice from day 4 after DSS exposure (Figure 1D). With regard to colon length, LFD+DSS caused shorter colons compared with WD+DSS treatment. Overall there was a significantly shorter colon length due to both diet (*P* < 0.0001) and DSS (*P* < 0.0001) (Figure 1E).

#### Levels of proinflammatory cytokines and LCN2 were increased in LFD-fed mice

**FIGURE 2:**
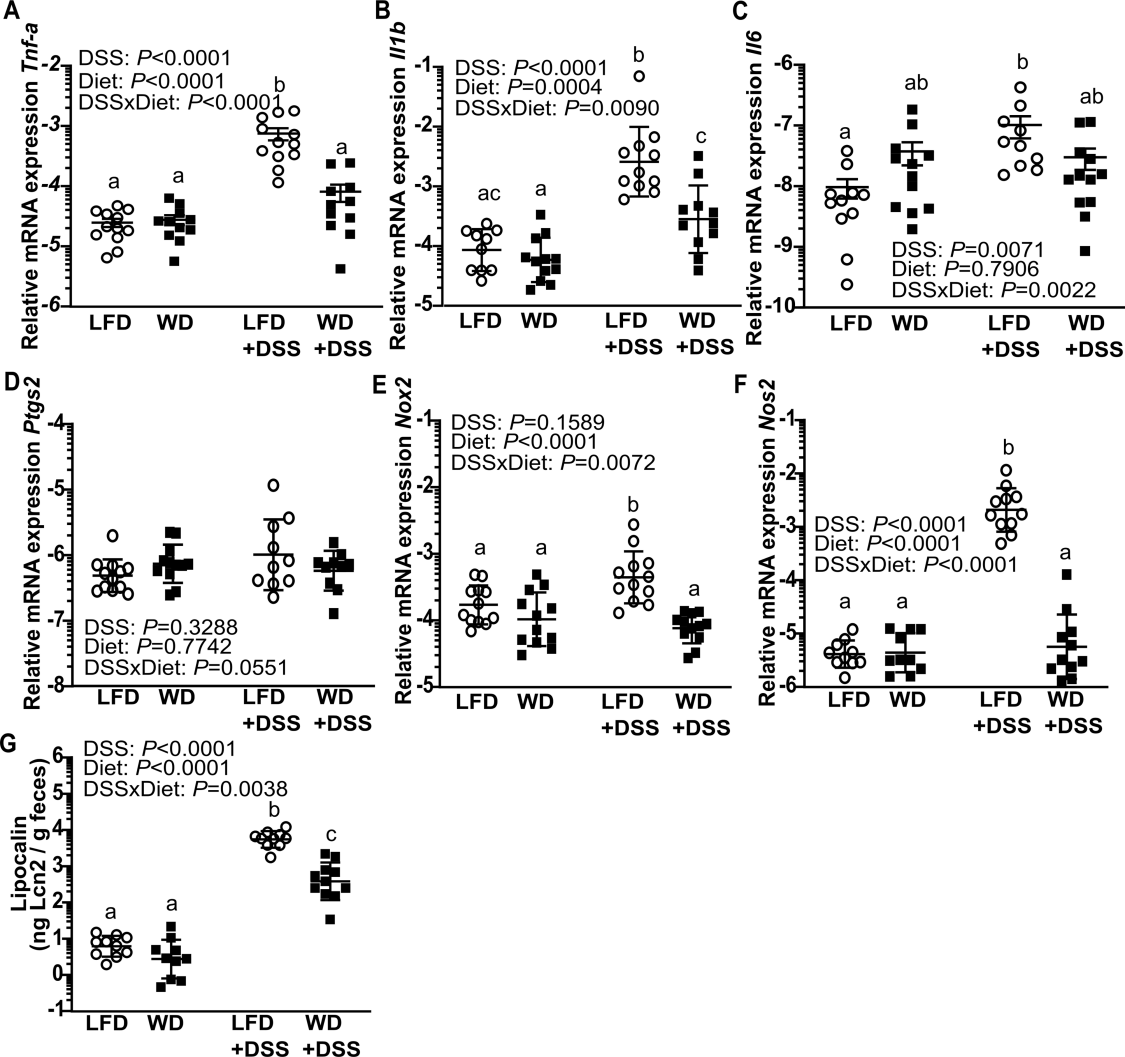
Relative mRNA expression of tumor necrosis factor α (*Tnf-a*) (A), interleukin-1 beta (*Il1b*) (B), interleukin 6 (*Il6*) (C), prostaglandin-endoperoxide synthase 2 (*Ptgs2*) (D), NADPH oxidase 2 (*Nox2*) (E), and nitric oxide synthase 2 (*Nos2*) (F) in mucosa from the proximal colon of LFD-fed and WD-fed mice with or without 1% DSS. Concentration of LCN2 in feces (G). Values are means ± SEM (*n* = 9–12). Labeled means without a common letter differ, *P* < 0.05. DSS, dextran sodium sulfate; LCN, lipocalin; LFD, low-fat diet; WD, Western diet.

The expression of the inflammatory genes, tumor necrosis factor alpha (*Tnf*-a), interleukin 1 beta (*Il1b*), interleukin 6 (*Il6*), and prostaglandin-endoperoxide synthase 2 (*Ptgs2*), in the distal colon was compared between the diet groups with and without DSS treatment (**Figure 2**A–D). For all genes except *Ptgs2* an interaction effect was found between diet and treatment (*P* < 0.01). In untreated mice, no differences were found between WD and LFD groups whereas DSS treatment led to a significant upregulation of these genes in the LFD+DSS mice compared with WD+DSS mice (*P* < 0.05). In WD-fed mice the expression levels of the above mentioned genes were not affected by DSS.

The same pattern of treatment × diet interaction was also observed for nitric oxide synthase 2 (*Nos2*) (also known as *iNos*) and NADPH oxidase 2 (*Nox2*), which was significantly higher in LFD+DSS mice (*P* < 0.001) compared with WD+DSS mice (Figure 2E,F). Finally we assessed the concentrations of LCN2 in feces, a sensitive marker of colonic inflammation. In both diet groups DSS treatment led to an increase of fecal LCN2, but the effect was significantly higher (*P* < 0.001) in LFD+DSS mice compared with WD+DSS mice (Figure 2G), also suggesting an interaction between treatment and diet for LCN2.

#### Gene expression for gut permeability was affected in LFD-fed mice

In addition to inflammatory related genes, expression of genes related to gut barrier and pattern recognition receptors was examined. DSS treatment led to a higher expression of toll-like receptor 4 (*Tlr4*), zonula occludens-1 (*Zo1*), and nucleotide-binding oligomerization domain 2 (*Nod2*) in the LFD mice when compared with WD-fed animals (*P* < 0.001).

**FIGURE 3:**
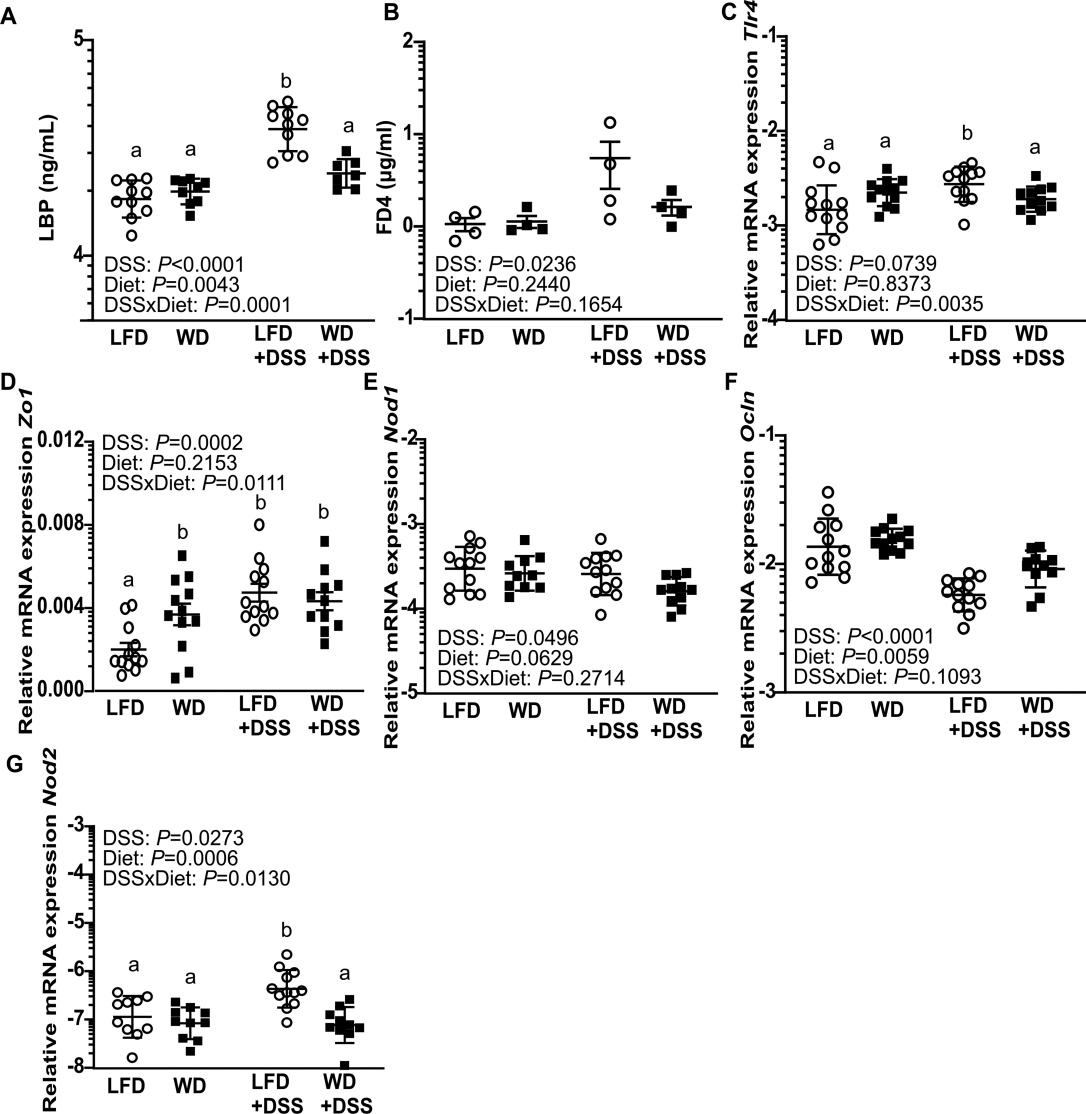
Concentration of LBP in plasma from LFD-fed and WD-fed mice with or without 1% DSS (A). FD4 in plasma 2 h after oral gavage (B). Relative mRNA expression of toll-like receptor 4 (*Tlr4*) (C), zonula occludens-1 (*Zo1*) (D), nucleotide-binding oligomerization domain 1 (*Nod1*) (E), occludin (*Ocln*) (F), and nucleotide-binding oligomerization domain 1 (*Nod2*) (G) in mucosa from the proximal colon of LFD-fed and WD-fed mice with or without 1% DSS. Values are means ± SEM (*n* = 7–12 apart from FD4 assay where *n* = 4). Labeled means without a common letter differ, *P* < 0.05. DSS, dextran sodium sulfate; FD4, FITC (fluorescein isothiocyanate) dextran 4 kDa; LBP, lipopolysaccharide binding protein; LFD, low-fat diet; WD, Western diet.

To investigate potential breach in the gut barrier, we assessed concentrations of lipopolysaccharide-binding protein (LBP) in plasma, which is an indicator of LPS leakage from the gut. A significantly higher concentration of LBP was found in LFD mice with DSS compared with WD mice with DSS (**Figure 3**A). The permeability of the gut influenced by diet and DSS, was further examined by assessment of plasma FD4 in 4 randomly selected mice per diet. We observed that DSS significantly increased plasma concentrations of FD4 (*P* = 0.024), but found no difference between the diet groups (Figure 3B).

When comparing untreated LFD and WD mice for *Tlr4* and *Zo1* mRNA abundance we observed that WD caused a higher expression of both these genes compared with LFD mice (*P* < 0.05) (Figure 3C,D). However, DSS treatment increased abundance of *Tlr4*and*Zo1* only in LFD mice (*P* < 0.001) and not in WD mice. Expression levels of *Nod1* and occludin (*Ocln*) genes were marginally downregulated by DSS treatment (*P* < 0.05), but no differences were noted between the 2 diet groups (Figure 3E,F).

#### DSS treatment caused a marked change in microbiota composition of LFD-fed mice

**FIGURE 4:**
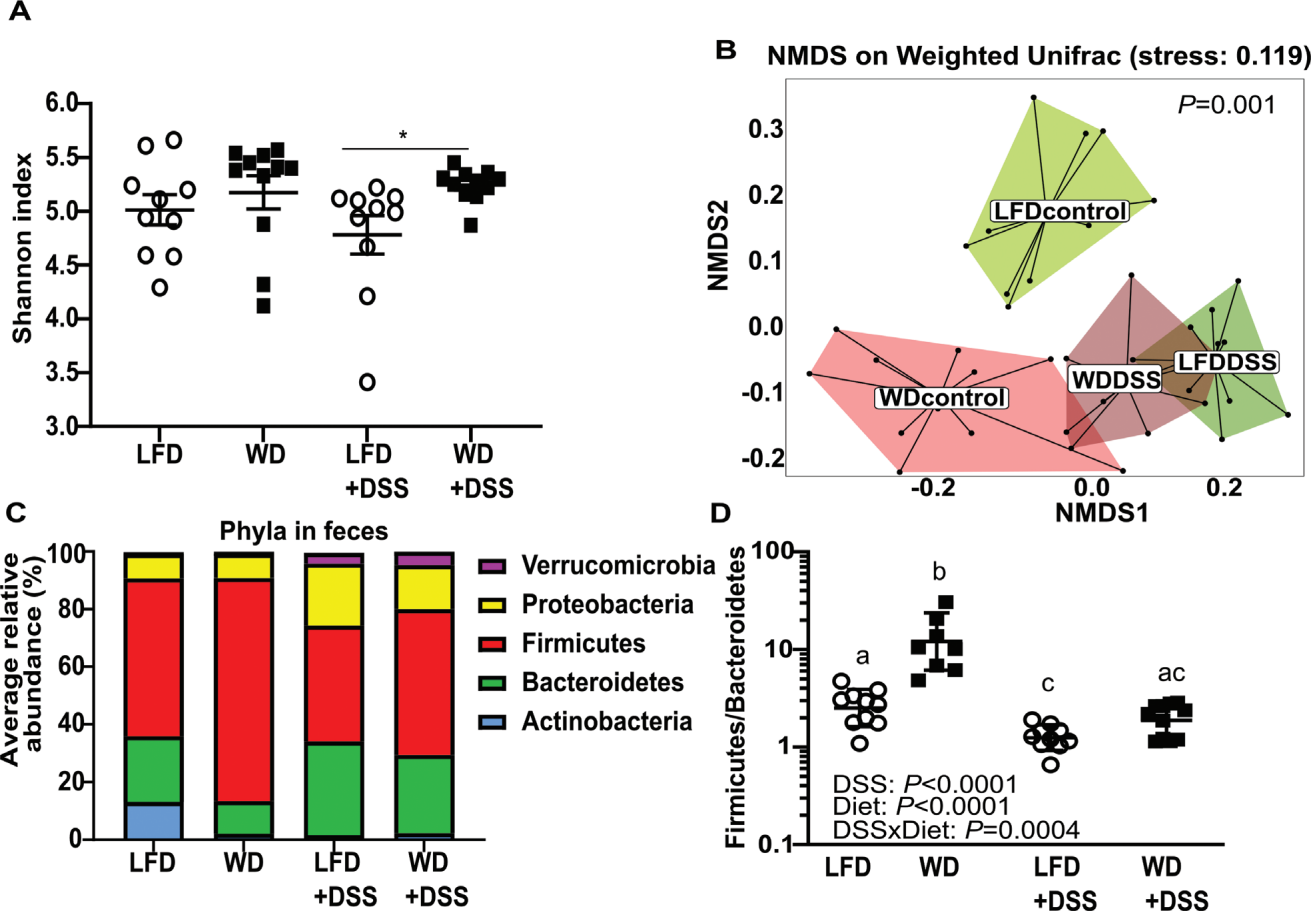
Microbiota analyses in feces from LFD-fed and WD-fed mice with or without 1% DSS. α Diversity with Shannon index (A), and β diversity with nonmetric multidimensional scaling (NMDS) of weighted UniFrac distances between groups (B). Colors indicate which group individual samples belong to (LFD control, WD control, LFD+DSS, WD+DSS). *P* = 0.001 in the 2-dimensional representation plot is from global PERMANOVA. Average relative abundance for all detected phyla for each group in fecal samples (C). *Firmicutes*/*Bacteroidetes* ratio in feces (D). Values are expressed as means ± SEM (*n* = 9–11). *Statistically significant difference, *P* < 0.05. Labeled means without a common letter differ, *P* < 0.05. DSS, dextran sodium sulfate; LFD, low-fat diet; PERMANOVA, permutational multivariate analysis of variance; WD, Western diet.

16S rRNA sequencing was performed on fecal pellets to elucidate differential effects of diets and DSS treatment on microbiota. LFD+DSS mice had a lower α diversity (within-sample diversity) compared with WD+DSS mice (*P* = 0.0006), whereas in untreated mice, no significant difference was found between the diet groups (**Figure 4**A).

β Diversity (between-sample diversity) showed significant differences between groups (Figure 4B). The LFD control group (untreated mice) was more diverse than the other groups, whereas the LFD+DSS mice were more similar to the WD control (untreated) and WD+DSS mice. The 5 most abundant phyla (relative average abundance >0.5%) were compared between all groups (Figure 4C). As illustrated by the *Firmicutes/Bacteroidetes* ratio (Figure 4D), untreated LFD-fed mice showed a higher abundance of the phylum *Bacteroidetes* and lower abundance of *Firmicutes* than untreated WD-fed mice. Notably untreated LFD-fed mice had high abundance of *Actinobacteria*, which was hardly detected in WD-fed mice. Abundance of *Proteobacteria* was similar in LFD- and WD-fed mice. Following 1% DSS administration, the abundance of *Proteobacteria* increased in both groups compared with untreated mice and a slight increase in *Bacteroidetes* and *Verrucomicrobia* was observed. *Firmicutes*, however decreased in abundance after DSS administration but with slightly higher levels in WD mice. *Actinobacteria* phylum was almost eliminated in LFD+DSS mice.

**FIGURE 5:**
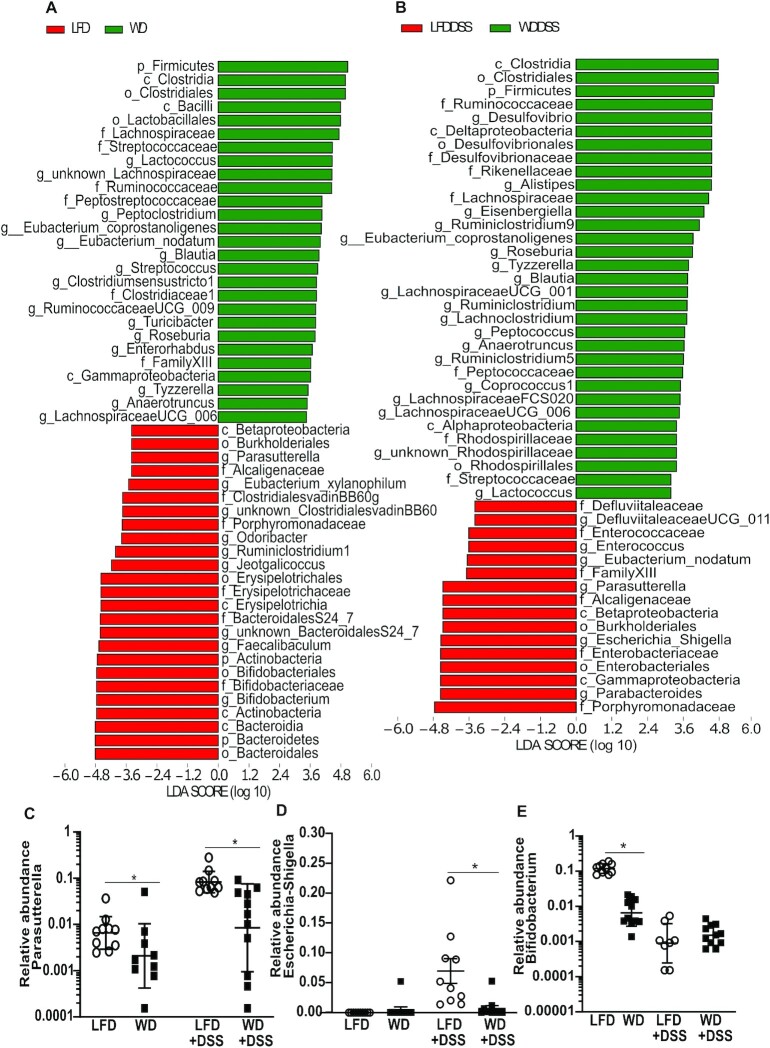
Comparison of the operational taxonomic units using linear discriminant effect size analysis and genera presence from *Actinobacteria* and *Proteobacteria* between the LFD-fed and WD-fed mice with or without 1% DSS. The histograms (A, B) present the taxa that explain the greatest differences between the LFD-fed and WD-fed mice untreated and treated with 1% DSS. Relative abundance of *Parasutterella* (C), *Escherichia-Shigella* (D), and *Bifidobacterium* (E) (*n* = 8–11). In panels C–E, *statistically significant difference, *P* < 0.05. c, class; DSS, dextran sodium sulfate; f, family; g, genus; LFD, low-fat diet; o, order; p, phylum; WD, Western diet.

LEfSe analyses (49) for non–DSS-treated and DSS-treated animals (**Figure 5**A,B) showed that genera belonging to the *Proteobacteria* phylum, such as *Parasutterella* and *Escherichia-Shigella*, increased significantly (*P* < 0.05) (Figure 5C,D) and there was a striking reduction of the genus *Bifidobacterium* (Figure 5E) in LFD mice treated with 1% DSS.

### Exp.2

#### WD reduced DSS inflammation regardless of fat source

**FIGURE 6:**
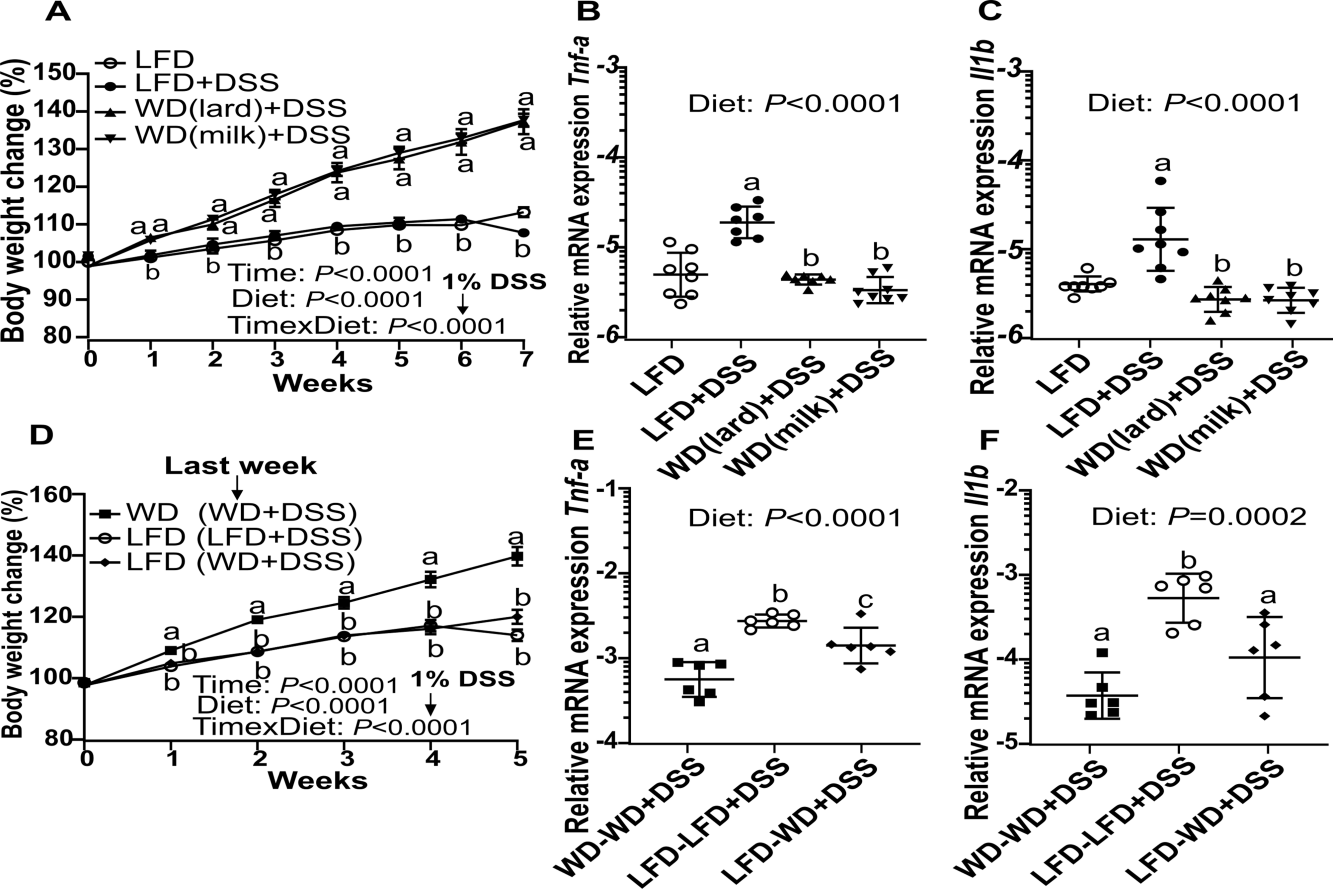
Body weight development (A) comparing the effects on 1% DSS treatment between the groups receiving WD_milkfat_, WD_lard_, or LFD (Exp.2). Relative mRNA expression of tumor necrosis factor α (*Tnf-a*) (B) and interleukin-1 beta (*Il1b*) (C) in mucosa from the proximal colon was compared between the groups (Exp.2). Values are means ± SEM (*n* = 8). Body weight development (D) comparing the group that changed from LFD into WD in the last week before DSS treatment and the groups that continued on LFD or WD (Exp.3). Relative mRNA expression of *Tnf-a* (E) and *Il1b* (F) in mucosa from the proximal colon were compared between the groups (Exp.3). Values are means ± SEM (*n* = 6). Labeled means without a common letter differ, *P* < 0.05. DSS, dextran sodium sulfate; Exp., Experiment; LFD, low-fat diet; WD, Western diet; WD_lard_, Western diet with lard fat; WD_milkfat_, Western diet with milk fat.

To investigate whether fat source was important for reducing DSS-mediated inflammation we compared LFD+DSS mice with WD+DSS mice where either milk fat or lard was used as the fat source in the WD (Exp.2). The results revealed similar protection against 1% DSS in both WD groups compared with LFD+DSS, regardless of fat source. As in Exp.1, the LFD+DSS mice showed the same pattern of weight loss (**Figure 6**A) and strong upregulation of *Tnf-a* and *Il1b* gene expression compared with WD+DSS containing either milk fat or lard as the fat source (Figure 6B,C).

### Exp.3

#### WD rapidly attenuated DSS-mediated inflammation

To test whether a WD offered an immediate “rescue effect” independent of long-term WD feeding, a third experiment was conducted (Exp.3). We here switched the diet from LFD to WD 1 d before applying DSS (LFD-WD+DSS) and compared this group with 2 other groups that were kept on the same diet from the start to the end of the experiment (WD-WD+DSS and LFD-LFD+DSS). WD introduced to LFD mice just prior to DSS treatment partially attenuated the DSS-mediated effects, both with regard to change in body weight (Figure 6D) and expression of *Tnf-a* and *Il1b* (Figure 6E,F). In line with the outcomes from Exp.1 and Exp.2, LFD-LFD+DSS mice experienced more weight loss and greater increase in proinflammatory genes compared with both WD groups (*P* < 0.05).

## Discussion

In the present study we investigated the effects of a WD on colon health and microbiota composition with and without a low-grade inflammation induced by 1% DSS. The main aim was to compare the effects of a WD with an LFD in mice. The 2 diets differed primarily in fat content and cholesterol. The main findings were that WD-fed mice were markedly less affected by DSS treatment compared with LFD-fed mice, which displayed a significantly increased degree of inflammation and had a microbiota composition deviating from that of non-DSS LFD mice.

Based on numerous reports on the detrimental effects of HFDs on intestinal health we initially hypothesized that WD would intensify colonic inflammation induced by DSS when compared with mice fed a LFD. It was therefore unexpected that the WD-fed group was only weakly affected by the DSS treatment whereas LFD mice were severely affected. This was shown not only when assessed clinically but also by using various inflammatory markers including proinflammatory gene expression, biomarker in feces (LCN2), and barrier integrity.

Our results hence contrast with most studies that have investigated the impact of fat-rich diets on DSS-induced colitis, which overall demonstrate that HFDs exacerbate DSS-induced colitis (22, 24, 26). However, in most of these experiments higher DSS concentrations were used and the results might therefore not be directly comparable.

Moreover, most of the studies finding adverse effects of WDs or HFDs, have used standard LFDs rich in dietary fiber as low-fat controls (unpurified diets). Such diets are poorly matched with the commonly used purified HFDs or WDs, which use the metabolically inert cellulose as the fiber source. The high diversity of fiber in unpurified diets, therefore, represents a confounder when interpreting results regarding effects of WDs or HFDs. Thus, it is possible that the adverse effect of WDs or other HFDs seen in many studies could be the result of a diet devoid of dietary fiber, which creates both a less diverse bacterial composition and blooming of bacteria that weakens the intestinal barrier (50) and not the high-fat content per se. In a study by Miles et al. (51), mice fed an unpurified diet were significantly more protected against DSS than mice fed a synthetic LFD.

Although our results contradict most studies investigating the impact of a WD, Enos and coworkers (52) demonstrated that mice fed a WD had significantly less tumor burden and inflammation in an AOM/DSS model of colorectal cancer compared with an LFD. The authors suggested that the protection against inflammation in that model could be ascribed to a higher content of mucin 2 (Muc2), which is the dominant protein in the protective mucus layer, and thereby strengthens the intestinal barrier . However, we did not find any difference in *Muc2* mRNA expression between LFD or WD mice (not shown).

Despite a more severe clinical impact of DSS in LFD-fed mice compared with WD, we found no differences in colonic inflammation between the 2 groups that were not exposed to DSS. However, WD-fed mice not exposed to DSS manifested increased expressions of *Zo1* and *Tlr4*, changes that could potentially strengthen the gut barrier and integrity. Tlr4 is a pathogen-recognition receptor and is important for eliciting downstream responses that maintain gut homeostasis (53, 54). Although permeability, as measured by FD4 leakage from gut to the blood, was unchanged in the LFD compared with the WD group, we cannot rule out that upregulation of *Zo1* and *Tlr4* are beneficial responses induced by WD for creating a more robust intestinal wall.

In terms of microbiota composition, we observed no difference in α diversity between LFD and WD in non–DSS-treated mice, but did notice a substantial difference in community structure (β diversity). In agreement with other studies (55, 56), we observed an increased *Firmicutes*/*Bacteriodetes* ratio in WD-fed mice. Interestingly, we also found a strikingly higher abundance of *Bifidobacteria* (phylum *Actinobacteria*) in LFD-fed mice prior to DSS treatment, which has also been reported by others (57). After DSS treatment, the abundance of *Bifidobacteria* dramatically decreased. Both the initial high level of *Bifidobacteria* and the sudden shift in abundance during DSS treatment could be possible drivers of the inflammatory process in the current experiment.

*Bifidobacteria* are generally considered beneficial commensals and are exploited for probiotic purposes (58, 59). Interestingly, a recent report found that supplementing mice with *Bifidobacteria* could protect against DSS-induced colitis, which argues against an unbeneficial effect of high pre-DSS levels in the LFD mice (60). However, certain strains of *Bifidobacteria* can promote intestinal inflammation through T helper 17 cells in the lamina propria (61). In this study, we did not detect any increase in other proinflammatory markers in LFD compared with WD in non–DSS-treated mice. Therefore, our results do not suggest that the higher *Bifidobacteria* abundance in the LFD before DSS treatment negatively influenced colitis development. Rather, we argue that the sudden shift in the abundance of *Bifidobacteria* during DSS treatment in the current study is a more likely explanation for the colitis development. Considering that *Bifidobacteria* are strict anaerobes, this genus is vulnerable to increased oxygen content in the gut following DSS treatment (62). In line with this argument, we found that expression of genes involved in production of reactive oxygen species, *Nox2* and *Nos2*, was upregulated in LFD+DSS mice.

*Proteobacteria* phylum increased in abundance in LFD+DSS mice compared with the WD+DSS mice. This is in agreement with other studies showing that *Proteobacteria* can be an indicator of an inflammatory phenotype with disease potential (63). In the LFD+DSS mice we also noticed a significant rise of genera belonging to the *Proteobacteria* such as *Escherichia, Shigella*, and *Parasutterella*. These observations agree with a recent study, where different doses of DSS (1%, 2%, 3%) increased the abundance of the family *Enterobacteriaceae* (which includes *Escherichia* and *Shigella*) and depleted *Bifidobacteria* (64).

The mechanism of how DSS induces colitis is not entirely known but it appears that DSS molecules disrupt the epithelial layer resulting in increased colonic epithelial permeability (21). Because DSS is a water-soluble, negatively charged sulfated polysaccharide we speculate that a WD with its high-fat content could create a hydrophobic layer on the intestinal surfaces that interferes with DSS and thereby inhibits the inflammatory action of DSS. To test whether WD had a direct effect on DSS, we performed a follow-up experiment in LFD mice switching the diet to WD just prior to DSS treatment. The WD given concomitantly with the DSS treatment protected against the DSS-induced colitis but the mice that were fed WD throughout the whole experiment were more protected. To the best of our knowledge we cannot find studies supporting that ingested fat can interfere with or neutralize induction of inflammation due to DSS. On the contrary, a study has shown that medium-chain fatty acids can chemically interact with DSS but lead to aggravated effects instead of a reduced colitis (65). There could also be other factors that interfere with establishing the DSS colitis. As suggested by Nell et al. (66) the induction of DSS-induced colitis depends on different factors, such as mouse strain, age, gender, body weight, lot number, molecular weight, concentration, and duration of exposure. To test whether the LFD mice had higher intake of DSS we also assessed water consumption, but found no difference between WD and LFD mice.

An alternative explanation for the observed effect of WDs is the influence of cholesterol (0.2%), which was added to the WD but not the LFD. Dietary cholesterol influences cholesterol homeostasis and leads to increased secretion of both free cholesterol and bile acids in the feces (67). Although we did not measure bile acids in this study, we can assume that concentrations of secondary bile acids in the colon were increased, with a potential impact on both microbiota composition and colonic health. Indeed concentrations of secondary bile acids in feces correlated with exacerbated DSS-induced colitis in mice (68), whereas in another study, secondary bile acids protected against DSS-induced colitis (69). Hence, based on the latter study we cannot rule out that cholesterol can in fact mediate some of the anti-inflammatory effects we observed.

In conclusion, our data demonstrate that a WD reduced DSS-induced colonic outcomes compared with an LFD regardless of whether the fat source was milk or lard. Although these data are somewhat conflicting with the general consensus that a WD adversely affects intestinal health, most previously reported experiments on this subject have rarely used LFD controls that match fiber content in the diet. Whether the protection against DSS is caused by a potential positive contribution of fat in WD or by other nutrients such as cholesterol should be further investigated. It is also possible that the DSS colitis mouse model, despite its popularity due to its rapidity, simplicity, and controllability, is not optimal to investigate the effects of HFDs on the development of colitis.

## Supplementary Material

nxab401_Supplemental_File

## Notes

Supported by the Norwegian Research Council, Bionær-#267858, and Norwegian University of Life Sciences (PhD grant).

Author disclosures: The authors report no conflicts of interest.

Supplemental Tables 1–11 are available from the “Supplementary data” link in the online posting of the article and from the same link in the online table of contents at https://academic.oup.com/jn/.

Abbreviations used: AOM, azoxymethane; DAI, disease activity index; DSS, dextran sodium sulfate; Exp., Experiment; FD4, FITC (fluorescein isothiocyanate) dextran 4 kDa; HFD, high-fat diet; *Il1b*, interleukin-1 beta; *Il6*, interleukin 6; LBP, lipopolysaccharide binding protein; LCN, lipocalin; LEfSe, linear discriminant analysis effect size; LFD, low-fat diet; Muc2, mucin 2; NGAL, neutrophil gelatinase-associated lipocalin; *Nod*, nucleotide-binding oligomerization domain; *Nos2*, nitric oxide synthase 2; *Nox2*, NADPH oxidase 2; PERMANOVA, permutational multivariate analysis of variance; *Ptgs2*, prostaglandin-endoperoxide synthase 2; QIIME, Quantitative Insights Into Microbial Ecology; *Tlr4*, toll-like receptor 4; *Tnf-a*, tumor necrosis factor alpha; WD, Western diet; *Zo1*, zonula occludens-1.
