# Supplementary material for: A High-Fat Western Diet Attenuates Intestinal Changes in Mice with DSS-Induced Low-Grade Inflammation
Source: J Nutr. 2021 Dec 2;152(3):758–69. doi: 10.1093/jn/nxab401 (PMC8891187; doi:10.1093/jn/nxab401)
Supplement: nxab401_Supplemental_File [file nxab401_supplemental_file.docx]

**“A high-fat Western diet attenuates intestinal changes in mice with DSS-induced low-grade inflammation”, Papoutsis et al**

**Online Supplementary Material**

# **SUPPLEMENTARY TABLE 1**

Compositions of experimental diets

|  | | | | |
| --- | --- | --- | --- | --- |
| Diets | **LFD** | | **WD** | |
| Ingredients | **g** | **kcal** | **g** | **kcal** |
| Casein | 195 | 780 | 195 | 780 |
| DL-methionine | 3 | 12 | 3 | 12 |
| Corn starch | 695.38 | 2780 | 50 | 200 |
| Maltodextrin 10 | 150 | 600 | 100 | 400 |
| Sucrose | 0 | 0 | 341 | 1364 |
| Milk fat, cow, anhydrous | 42.5 | 320 | 200 | 1800 |
| Corn oil | 10 | 90 | 10 | 90 |
| Cellulose, BW200 | 70 | 0 | 70 | 0 |
| Ethoxyquin | 0.04 | 0 | 0.04 | 0 |
| Mineral mix S10001 | 35 | 0 | 35 | 0 |
| Calcium carbonate | 4 | 0 | 4 | 0 |
| Vitamin mix V10001 | 10 | 40 | 10 | 40 |
| Choline bitartrate | 2 | 0 | 2 | 0 |
| Cholesterol | 0 | 0 | 1.5 | 0 |
| Total | 1216.9 | 4592.4 | 1021.5 | 4592.4 |
| Protein, % | 14.3 | 15.2 | 17.1 | 15.2 |
| Carbohydrate, % | 70.2 | 74.5 | 49 | 43.6 |
| Fat, % | 4.3 | 10.3 | 20.1 | 41.2 |
| kcal/g | 3.8 |  | 4.5 |  |

LFD, low fat diet (D14042701, Research Diets); WD, Western diet (D12079B, Research Diets).

# **SUPPLEMENTARY TABLE 2**

Fatty acid profile of fat sources.

| **Fat source** | **Milk** | **Lard** |
| --- | --- | --- |
| Ingredients | g | g |
| Butter, Anhydrous | 200 | 0 |
| Corn Oil | 10 | 10 |
| Lard |  | 200 |
| Total | 210 | 210 |
|  |  |  |
| Fatty Acid | g | g |
| C4, Butyric | 6.4 | 0.0 |
| C6, Caproic | 3.8 | 0.0 |
| C8, Caprylic | 2.2 | 0.0 |
| C10, Capric | 5.0 | 0.1 |
| C12, Lauric | 5.6 | 0.2 |
| C14, Myristic | 20.0 | 2.3 |
| C14:1, Myristoleic | 3.0 | 0.0 |
| C15, Pentadecanoic | 0.0 | 0.2 |
| C16, Palmitic | 53.5 | 39.7 |
| C16:1, Palmitoleic | 4.6 | 2.7 |
| C17, Heptadecanoic | 0.0 | 0.7 |
| C18, Stearic | 24.4 | 21.4 |
| C18:1, Oleic | 52.7 | 68.2 |
| C18:2, Linoleic | 10.6 | 54.8 |
| C18:3, Linolenic | 2.9 | 2.8 |
| C20, Arachidic | 1.9 | 0.3 |
| C20:1, Eicosenoic | 0.0 | 1.2 |
| C20:2, Eicosadienoic | 0.0 | 1.6 |
| C20:3, Dihomo-gamma-linolenic | 0.0 | 0.3 |
| C20:4, Arachidonic | 0.0 | 0.6 |
| C22:5, Docosapentaenoic | 0.0 | 0.2 |
| Total | 196.6 | 197.3 |
| Saturated | 122.8 | 64.9 |
| Monounsaturated | 60.3 | 72.2 |
| Polyunsaturated | 13.6 | 60.2 |
|  |  |  |
|  | % (wt: wt) | % (wt: wt) |
| Saturated | 62.4 | 32.9 |
| Monounsaturated | 30.7 | 36.6 |
| Polyunsaturated | 6.9 | 30.5 |

Wt, weight.

# **SUPPLEMANTARY TABLE 3**

Criteria for DAI and scoring way for assessing during exposure to 1% DSS.

| **For weight** |
| --- |
| 0=<5% weight loss |
| 1= 5-15% weight loss |
| 5= 15-20% weight loss |
| 10=> 20% weight loss |

Score given regarding body weight loss:

Score given regarding mouse welfare:

# **SUPPLEMENTARY TABLE 4**

Reaction mixture for cDNA synthesis using the iScript cDNA Synthesis kit (Bio Rad).

| **Component** | **Per reaction** |
| --- | --- |
| 5x iScript reaction mix | 4 µL |
| iScript reverse transcriptase | 1 µL |
| Nuclease-free water | 11 µL |
| RNA template (200ng/µL) | 4 µL |

# **SUPPLEMENTARY TABLE 5**

Temperature program used for cDNA synthesis.

| **Operation** | **Temperature (°C)** | **Duration** |
| --- | --- | --- |
| Primer annealing | 25 | 5 min |
| cDNA synthesis | 42 | 30 min |
| cDNA synthesis termination | 85 | 5 min |
| - | 4 | ∞ |

# **SUPPLEMENTARY TABLE 6**

Primer sequences for RT-qPCR and melting temperatures

| **Gene** | **Forward Primer 5’-3’** | **Reverse Primer 5’-3’** | **Tm °C** |
| --- | --- | --- | --- |
| *Gapdh* | CTTCAACAGCAACTCCCACTCTT | GCCGTATTCATTGTCATACCAGG | 60 |
| *Il1b* | GCAGCTGGAGAGTGTGGAT | AAACTCCACTTTGCTCTTGACTT | 61 |
| *Il6* | CGTGGAAATGAGAAAAGAGTTGT | GCATCATCGTTGTTCATACA | 61 |
| *Nod1* | TGACAGTAATCTGGCTGACC | GTCTGGTTCACTCTCAGCAT | 59 |
| *Nod2* | GCAGAAACTAGCTCTCTTCAAC | CGGCTGTGATGTGATTGTTC | 61 |
| *Nos2* | GACATTACGACCCCTCCCAC | ACTCTGAGGGCTGACACAAG | 62 |
| *Nox2* | GGGAACTGGGCTGTGAATGA | CAGTGCTGACCCAAGGAGTT | 61 |
| *Ocln* | CTGTGAAAACCCGAAGAAAGATG | GCAGACACATTTTTAACCCACTC | 57 |
| *Ptgs2* | AATATCAGGTCATTGGTGGAGA | TCTACCTGAGTGTCTTTGACTG | 61 |
| *Tlr4* | GATCTGAGCTTCAACCCCTT | TGTTTCAATTTCACACCTGGA | 61 |
| *Tnfa* | CTGTCTACTGAACTTCGGGGTGAT | GGTCTGGGCCATAGAACTGATG | 61 |
| *Zo1* | GAGAAAGGTGAAACTCTGCTG | ACGAGGAGTCGGATGATTTTAGA | 59 |
|  |  |  |  |

**Abbreviations:** *Gapdh***,** glyceraldehyde 3-phosphate dehydrogenase; *Il1b*, interleukin-1 beta; *Il6*, interleukin 6; *Nod1*, nucleotide-binding oligomerization domain 1; Nod2, nucleotide-binding oligomerization domain 2; *Nos2*, nitric oxide synthase 2; *Nox2*, NADPH oxidase 2; *Ocln*, Occludin; *Ptgs2*, prostaglandin endoperoxide synthase 2; *Tlr4*, toll like receptor 4; *Tnf*a, tumor necrosis factor alpha; *Zo1*, Zonula occludens 1.

# **SUPPLEMENTARY TABLE 7**

Reaction mixture for amplicon PCR during library preparation for gene sequencing of 16S rRNA.

| **Component** | **Per reaction** |
| --- | --- |
| 5x HOT FIREPol® Blend Master Mix Ready to Load (Solis BioDyne) | 5 µL |
| Forward primer, PRK341F (1 µM)* | 0.5 µL |
| Reverse primer, PRK806R (1 µM)* | 0.5 µL |
| Nuclease-free water | 18 µL |
| Template DNA (0.003-2 ng/µL**) | 1 µL |

* Forward 5’- CCTACGGGRBGCASCAG-3’, reverse 5’- GGACTACYVGGGTATCTAAT-3’
** Measured by Qubit.

# **SUPPLEMENTARY TABLE 8**

Temperature cycles for amplicon PCR during library preparation for gene sequencing of 16S rRNA.

| **Operation** | **Temperature (°C)** | **Duration** | **Cycles** |
| --- | --- | --- | --- |
| Initial activation | 95 | 15 min | 1 |
| Denaturation | 95 | 30 sec | 25 |
| Annealing | 55 | 30 sec |  |
| Elongation | 72 | 45 sec |  |
| Final elongation | 72 | 7 min | 1 |
| - | 4 | ∞ | - |

# **SUPPLEMENTARY TABLE 9**

Reaction mixture for index PCR during library preparation for gene sequencing of 16S rRNA.

| **Component** | **Per reaction** |
| --- | --- |
| 5x FIREPol® Master Mix Ready to Load (Solis BioDyne) | 5 µL |
| Forward primer (1 µM)* | 5 µL |
| Reverse primer (1 µM)* | 5 µL |
| Nuclease-free water | 8 µL |
| Template DNA | 2 µL |

* See Supplementary table 11

# **SUPPLEMENTARY TABLE 10**

Temperature cycles for index PCR during library preparation for gene sequencing of 16S rRNA.

| **Operation** | **Temperature (°C)** | **Duration** | **Cycles** |
| --- | --- | --- | --- |
| Initial activation | 95 | 5 min | 1 |
| Denaturation | 95 | 30 sec | 10 |
| Annealing | 55 | 1 min |  |
| Elongation | 72 | 45 sec |  |
| Final elongation | 72 | 7 min | 1 |
| - | 4 | ∞ | - |

# **SUPPLEMENTARY TABLE 11**

Primers modified with Illumina adapters used for index PCR during library preparation for gene sequencing of 16S rRNA. Unique combination of forward and reverse primer was used for each sample.

| **Primer name** | **Sequence, 5' -> 3'** | **Target region** | **Direction** |
| --- | --- | --- | --- |
| **F9** | aatgatacggcgaccaccgagatctacactctttccctacacgacgctcttccgatctgtttcgCCTACGGGRBGCASCAG | 16S rRNA (V3-V4) | Forward |
| **F10** | aatgatacggcgaccaccgagatctacactctttccctacacgacgctcttccgatctcgtacgCCTACGGGRBGCASCAG | 16S rRNA (V3-V4) | Forward |
| **F11** | aatgatacggcgaccaccgagatctacactctttccctacacgacgctcttccgatctgagtggCCTACGGGRBGCASCAG | 16S rRNA (V3-V4) | Forward |
| **F12** | aatgatacggcgaccaccgagatctacactctttccctacacgacgctcttccgatctggtagcCCTACGGGRBGCASCAG | 16S rRNA (V3-V4) | Forward |
| **F13** | aatgatacggcgaccaccgagatctacactctttccctacacgacgctcttccgatctactgatCCTACGGGRBGCASCAG | 16S rRNA (V3-V4) | Forward |
| **F14** | aatgatacggcgaccaccgagatctacactctttccctacacgacgctcttccgatctatgagcCCTACGGGRBGCASCAG | 16S rRNA (V3-V4) | Forward |
| **F15** | aatgatacggcgaccaccgagatctacactctttccctacacgacgctcttccgatctattcctCCTACGGGRBGCASCAG | 16S rRNA (V3-V4) | Forward |
| **F16** | aatgatacggcgaccaccgagatctacactctttccctacacgacgctcttccgatctcaaaagCCTACGGGRBGCASCAG | 16S rRNA (V3-V4) | Forward |
| **R25** | caagcagaagacggcatacgagatATCAGTgtgactggagttcagacgtgtgctcttccgatctGGACTACYVGGGTATCTAAT | 16S rRNA (V3-V4) | Reverse |
| **R26** | caagcagaagacggcatacgagatGCTCATgtgactggagttcagacgtgtgctcttccgatctGGACTACYVGGGTATCTAAT | 16S rRNA (V3-V4) | Reverse |
| **R27** | caagcagaagacggcatacgagatAGGAATgtgactggagttcagacgtgtgctcttccgatctGGACTACYVGGGTATCTAAT | 16S rRNA (V3-V4) | Reverse |
| **R28** | caagcagaagacggcatacgagatCTTTTGgtgactggagttcagacgtgtgctcttccgatctGGACTACYVGGGTATCTAAT | 16S rRNA (V3-V4) | Reverse |
| **R29** | caagcagaagacggcatacgagatTAGTTGgtgactggagttcagacgtgtgctcttccgatctGGACTACYVGGGTATCTAAT | 16S rRNA (V3-V4) | Reverse |
| **R30** | caagcagaagacggcatacgagatCCGGTGgtgactggagttcagacgtgtgctcttccgatctGGACTACYVGGGTATCTAAT | 16S rRNA (V3-V4) | Reverse |
| **R31** | caagcagaagacggcatacgagatATCGTGgtgactggagttcagacgtgtgctcttccgatctGGACTACYVGGGTATCTAAT | 16S rRNA (V3-V4) | Reverse |
| **R32** | caagcagaagacggcatacgagatTGAGTGgtgactggagttcagacgtgtgctcttccgatctGGACTACYVGGGTATCTAAT | 16S rRNA (V3-V4) | Reverse |
| **R33** | caagcagaagacggcatacgagatCGCCTGgtgactggagttcagacgtgtgctcttccgatctGGACTACYVGGGTATCTAAT | 16S rRNA (V3-V4) | Reverse |
| **R34** | caagcagaagacggcatacgagatGCCATGgtgactggagttcagacgtgtgctcttccgatctGGACTACYVGGGTATCTAAT | 16S rRNA (V3-V4) | Reverse |
| **R35** | caagcagaagacggcatacgagatAAAATGgtgactggagttcagacgtgtgctcttccgatctGGACTACYVGGGTATCTAAT | 16S rRNA (V3-V4) | Reverse |
